# Rules for Shaping Neural Connections in the Developing Brain

**DOI:** 10.3389/fncir.2016.00111

**Published:** 2017-01-10

**Authors:** Elena Kutsarova, Martin Munz, Edward S. Ruthazer

**Affiliations:** ^1^Montreal Neurological Institute, McGill UniversityMontreal, QC, Canada; ^2^Friedrich Miescher Institute, Neurobiology GroupBasel, Switzerland

**Keywords:** retinotectal, visual system, colliculus, optic tectum, activity-dependent, Hebbian plasticity, review, retinal ganglion cell

## Abstract

It is well established that spontaneous activity in the developing mammalian brain plays a fundamental role in setting up the precise connectivity found in mature sensory circuits. Experiments that produce abnormal activity or that systematically alter neural firing patterns during periods of circuit development strongly suggest that the specific patterns and the degree of correlation in firing may contribute in an instructive manner to circuit refinement. In fish and amphibians, unlike amniotic vertebrates, sensory input directly drives patterned activity during the period of initial projection outgrowth and innervation. Experiments combining sensory stimulation with live imaging, which can be performed non-invasively in these simple vertebrate models, have provided important insights into the mechanisms by which neurons read out and respond to activity patterns. This article reviews the classic and recent literature on spontaneous and evoked activity-dependent circuit refinement in sensory systems and formalizes a set of mechanistic rules for the transformation of patterned activity into accurate neuronal connectivity in the developing brain.

## Introduction

In this review article, we propose a detailed set of cellular rules that govern activity-dependent circuit refinement. This list of rules synthesizes what has been learned in the extensive experimental literature on the development of the visual system, with a strong emphasis on data obtained from live imaging of the retinotectal projection in fish and frogs. Because of their external development and largely translucent bodies, permitting high-resolution *in vivo* imaging of developing neurons and their associated glial cells, albino tadpoles of the African clawed frog (*Xenopus laevis*) and larval zebrafish (*Danio rerio*) in particular have been popular models for studying activity-dependent circuit development and remodelling. Moreover, unlike mammals, these animals rely extensively on vision for survival from very early developmental stages, and use this same visual information to direct circuit refinement.

While molecular guidance cues are critical for establishing the initial crude topographic projections from the eye to the brain, even greater precision of neuronal maps is achieved through the involvement of activity-dependent mechanisms. Because neighboring retinal ganglion cells (RGCs) in the eye are more likely to exhibit temporal correlation in their firing patterns than distantly separated RGCs, the pattern of firing of action potentials in the developing visual system contains important information about the relative position of the RGC somata in the retina. The system therefore is able to use patterned neural activity in RGCs to instruct the orderly mapping of their axons onto postsynaptic partners in the optic tectum. This results in a more precise map of the retina onto the optic tectum. This review article will discuss classic studies and more recent experimental insights from *in vivo* imaging that reveal fundamental details about how activity-dependent structural and functional refinement takes place. The refinement of the retinotectal circuit achieves a remarkably accurate retinotopic representation that contributes to effective visual processing and ultimately to the generation of visually-guided behaviors. Here, we define refinement as the process of establishing precise anatomical wiring (i.e., the representation most closely reproducing the input space), which allows for the optimal function of neural circuits in the animal.

## Retinotopic Maps and Eye-Specific Segregation in the Developing Central Nervous System

In fish and frogs the RGCs project to at least 10 distinct tectal and pretectal arborization fields believed to mediate important behavioral responses such as eye movements and prey capture (Easter and Taylor, 1989; Burrill and Easter, 1994; Kubo et al., 2014; Semmelhack et al., 2014). By far, the most extensive projection terminates in the contralateral optic tectum, where the organization of axonal terminals reconstitutes a topographic map of the retina. The axons of RGCs whose somata are located in the temporal retina project to the rostral tectum, whereas RGCs residing in the nasal retina send their axons to the caudal tectum (Attardi and Sperry, 1963). Similarly, the dorsoventral axis of the retina is represented medio-laterally in the optic tectum. The retinocollicular projection in mammals forms a retinotopic map with a similar coordinate system in the analogous structure to the optic tectum, the superior colliculus (SC) (N.B., The term optic tectum is applied generically to represent the analogous retinorecipient structure in all vertebrates). The SC is involved in directing eye and head movements in mammals (Schiller, 1972).

Unlike amphibians, in which the retinofugal projection is almost exclusively contralateral, in mammals a fraction of the RGCs does not cross at the optic chiasm but projects to the ipsilateral hemisphere of the brain. This fraction ranges from 3% to 5% in mice, around 15% in ferrets to up to nearly 40% in humans (Petros et al., 2008). Interestingly, anterograde and retrograde tracing of RGCs also reveals a substantial transient ipsilateral projection during embryonic development in mammals and chicks, much of which is lost during subsequent maturation (Land and Lund, 1979; Dräger and Olsen, 1980; McLoon and Lund, 1982). In the mammalian brain, inputs from both eyes project to the deep part of the stratum griseum superficiale and to the stratum opticum of the SC. These binocular projections segregate into alternating eye-specific bands in the rostral colliculus (Godement et al., 1984). The more superficial part of the stratum griseum superficiale of the SC normally receives exclusively contralateral eye input. In addition to providing afferents to the SC, RGC axons also project to the visual thalamus in mammals (Sretavan and Shatz, 1984). A distinct visual processing pathway, the retinothalamic (retinogeniculate) projection terminates in both the ventral and dorsal lateral geniculate nuclei (LGN) in the thalamus. The dorsal LGN is thought to serve as the fundamental relay station through which visual information is passed to higher order cortical visual centers where increasingly complex features are extracted from visual scenes (Fellenman and Van Essen, 1991). The afferents in the LGN, much like in the SC, project retinotopically but segregate into eye-specific laminae (Linden et al., 1981).

This review article focuses primarily on the retinotectal projection and outlines the evidence supporting a specific set of rules for projection refinement based on activity-dependent cellular mechanisms involved in the establishment and refinement of the functional retinotopic map in the optic tectum. Experimental perturbations and live observations of labeled neurons in the developing brains of small transparent tadpoles and fish larvae constitute the main source of data that explain how map development proceeds at the cellular level. However, the same mechanisms responsible for retinotectal development in these simpler vertebrates are also likely to play essential roles in mammalian development. We have highlighted experiments from multiple species that have provided particularly important insights into projection refinement mechanisms throughout this review article. In addition, it is important to note that the most prominent activity-dependent stages of brain circuit refinement do not necessarily take place at the same time in development (e.g., the retinal projection to the SC achieves its mature organization earlier than that in the LGN) and therefore the rules that control retinotectal refinement may be fundamentally different, or manifest themselves differently, during later refinement events.

We begin by outlining a list of fundamental mechanistic events that the experimental evidence indicates are likely to occur during retinotectal map refinement. Each of these is elaborated in greater detail below.

## Rules for Retinotectal Structural Plasticity

1. Molecular guidance cues provide information for coarse axonal targeting.
2. Inputs compete for available synaptic target space.
3. Axonal and dendritic arbors are highly dynamic, even after seemingly mature morphology is attained.
4. Patterned neuronal activity provides instructive cues that help refine inputs:
  (a) Synchronous firing stabilizes synapses and prolongs branch lifetimes while actively suppressing branch dynamics via N-methyl D-aspartate receptor (NMDAR)-dependent retrograde signaling (Hebbian mechanisms).
  (b) Asynchronous activity weakens synapses (LTD) and actively promotes axonal branch dynamics, including addition and elongation, as well as branch elimination (Stentian mechanisms).

5. In the absence of sensory input, correlated spontaneous firing provides surrogate patterned activity.
6. New axonal branch tips emerge near existing synapses.
7. Stronger synapses help stabilize the axons and dendrites on which they form (Synaptotropism).
8. Homeostatic mechanisms help maintain the overall level of functional synaptic input to the target.

## 1. Molecular Guidance Cues Provide Information for Coarse Axonal Targeting

The classic experiments of Sperry (1963) studying the developing and regenerating retinotectal projection in fish and amphibians, led Sperry to propose the “Chemoaffinity Hypothesis” in which the molecular tagging of presynaptic and postsynaptic partners with a kind of address code guides the establishment of the retinotopic maps in the optic tectum. Sperry (1943) observed that following optic nerve section, regenerating RGC axons projected back to roughly correct, retinotopically appropriate sites in the optic tectum irrespective of whether the whole retina was still present, even if the eye had been rotated to provide erroneous information about the visual environment. Furthermore, even upon initial entry to the optic tectum, RGC inputs are already coarsely retinotopically distributed within the optic tract (Holt and Harris, 1983), suggesting the presence of molecular guidance cues throughout the developing visual system.

Concrete support for this model came initially with the identification of a candidate molecular activity expressed in membranes isolated from posterior optic tectal cells that could serve as one of Sperry’s tags (Walter et al., 1987). Ultimately the molecule was identified to be a ligand of the EphA family of receptor tyrosine kinases, and together with related EphA binding molecules, this group of ligands was labeled as the ephrin-A family (Drescher et al., 1995; Feldheim and O’Leary, 2010). In mammals, there are nine members of the EphA subclass (EphA1-EphA8 and EphA10) and five members of the EphB subclass (EphB1-EphB4 and EphB6), the molecular properties and signaling of which have been exhaustively reviewed (see Egea and Klein, 2007). Studies in numerous species have demonstrated that EphA and ephrin-A members are expressed in complementary gradients across the retina and the tectum (Brennan et al., 1997; Monschau et al., 1997; McLaughlin et al., 2003a; Higenell et al., 2012). The expression levels of EphA are highest in temporal retina and decline in a graded manner toward the nasal retina; A decreasing gradient of ephrin-A from the caudal to the rostral tectum is also present. Ephrin-A and EphA family members are expressed at the surface of both RGC axons and tectal cells. Once bound, the receptor-ligand couple activates a signaling pathway that results in axon repulsion (Drescher et al., 1997). Therefore, temporal axons expressing high EphA levels avoid the caudal tectum where the ephrin-A levels peak.

Interestingly, the complementary expression gradients of ephrins and Ephs mean that they are co-expressed at differing ratios within cells across the retina and the tectum (Marcus et al., 1996; Rashid et al., 2005). Ephrin-A5 is expressed in a strong graded fashion (nasal > temporal) in the mouse retina (Suetterlin and Drescher, 2014). Thus, cis-signaling mediated by Eph-ephrin binding within the same RGC axon may serve to effectively sharpen the nasotemporal gradient of trans-neuronal ephrin signaling (Hornberger et al., 1999). Moreover, the high levels of ephrin-A5 expressed on nasal axons allows them to also participate in the repulsion of temporal axons that express high levels of EphA (Bonhoeffer and Huf, 1980; Raper and Grunewald, 1990; Suetterlin and Drescher, 2014).

Just as the interaction of EphA and ephrin-A mediates the mapping of the temporal-nasal axis of the retina along the rostral-caudal dimension of the tectum, the interaction of EphB with ephrin-B has been proposed to contribute to the dorsoventral mapping of RGC terminals along the mediolateral axis of the optic tectum. However, the underlying interaction appears to mediate axonal attraction rather than repulsion and may also involve reverse signaling from EphB to ephrin-B (Hindges et al., 2002; Mann et al., 2002). While the importance of EphA-ephrin-A signaling in establishing the anteroposterior axis of the retinotectal topographic map has been well validated with knock-out and misexpression studies (Feldheim and O’Leary, 2010), there remains some uncertainty about the specific roles played by EphB-ephrin-B signaling. In frogs, the actual gradient of expression during development is not consistent with that predicted by the cell biology. The proposed reverse signaling model, based on *in vitro* observations, suggested that dorsal RGCs expressing ephrin-B should be attracted to putative high levels of EphB in the ventral tectum (Mann et al., 2002). However, labeling the expression pattern of EphB in the *Xenopus* tadpole brain using ephrin-B-alkaline phosphatase fusion proteins revealed that, to the contrary, there is instead a dorsal > ventral expression gradient of EphB and no detectable ephrin-B gradient in the optic tectum (Higenell et al., 2012). Furthermore, the dorsoventral axis of the retinofugal projection appears to presort in the optic tract prior to encountering any gradient in the tectum, suggesting that EphB-ephrinB signaling may have a role to play in axon guidance in the optic tract (Plas et al., 2005).

## 2. Inputs Compete for Available Synaptic Target Space

Aside from the chemoaffinity and chemorepulsive mechanisms that direct RGC axons to arborize roughly within topographically appropriate locations in the target, several lines of evidence indicate that the mapping of the projection is subject to an additional fundamental influence that is most likely independent of neural activity. There appears to be a competition for available target space that has the important consequence of rendering the retinotectal projection free of discontinuities and innervation gaps. One striking example of this phenomenon can be seen in the retinotectal projection of the ephrin-A2/A5 double knock-out mouse, which has severely disrupted mapping of RGC inputs along its rostrocaudal axis, such that injection of the anterograde tracer DiI into one site in the eye results in multiple discrete patches of axon terminal labeling along the entire axis instead of a focused single termination zone in the target as seen in wildtype animals (Feldheim et al., 2000). Despite the complete topographic disorganization of retinal inputs, bulk labeling the entire retinotectal projection by intraocular cholera toxin B injection produces a uniform and uninterrupted pattern of afferent labeling across the entire area of the tectal neuropil which is indistinguishable from that in wildtype animals. Thus the ability of the inputs to occupy all available target space is unperturbed despite the absence of the ephrin-A signaling that is essential for ordering the map.

An extreme version of this phenomenon, which offers some insights into the importance of this competition over target space in map formation is the so-called “EphA ki/ki” transgenic mouse in which two overlapping gradients of EphA expression are induced in the retina, one normal and one elevated by uniform high expression of EphA3 in a subset of RGCs (Islet2+ cells) throughout the eye (Reber et al., 2004). In these mice, RGCs expressing the normal levels of graded EphA project to the tectum and form a complete, topographically ordered map that is restricted to the caudal half of the tectum. In the rostral tectum a second complete and well-ordered map is formed by the Islet2+ RGCs. This double map reveals that in distinction to the pure chemoaffinity model put forth by Sperry, it is relative, rather than absolute, levels of EphA receptor expression that organize the map. The fact that two full maps form in the space that normally accommodates one further supports the idea that maps can expand or contract as necessary to fill the available territory.

In animals capable of central nervous system regeneration like fish and frogs it has long been known that if half the retina is ablated and the remaining half allowed to regrow into the tectum, the resulting half-map expands to fill the territory formerly occupied by afferents from the intact retina (Schmidt and Easter, 1978). Similarly, ablation of part of the tectum results in a compressed retinotectal map that fits within the remaining area (Yoon, 1976; Schmidt and Coen, 1995). It appears that this process of map regulation, by which input and target size are matched, may occur without the benefit of patterned neural activity, as injection of the sodium channel blocker tetrodotoxin (TTX) failed to prevent the map rescaling (Meyer and Wolcott, 1987). These observations support the notion of activity-independent competitive interactions in the optic tectum, perhaps analogous to the competition of peripheral axons for nerve growth factor in the skin (Lewin and Barde, 1996). The reorganization of the map that occurs after ablations therefore appears to represent an independent influence that the system imposes on the afferents through competition for space.

Map plasticity has also been examined at the single axon level in a clever experiment in zebrafish in which a single arbor from a transplanted RGC is allowed to innervate the optic tectum of a *lakritz* mutant fish that is incapable of generating its own RGCs. This single axon is free to innervate its target in the complete absence of competition from other retinal afferents (Gosse et al., 2008). Interestingly, these axons managed to target their topographically appropriate termination zones in the tectum, but formed abnormally large terminal arbors. This result suggests that retinal axons do at least have a crudely defined inherent preferred termination zone within the target, presumably due to chemoaffinity cues, but that in the absence of competition for space, arbors can enlarge their coverage area, at least to a limited extent.

Is there a role for neural activity in the competition between afferents? This question was addressed in an experiment in which the Kir2.1 potassium channel which reduces neuronal excitability and firing was overexpressed in just a few RGCs together with GFP in zebrafish larvae (Hua et al., 2005). These silenced RGC axons failed to elaborate arbors in the optic tectum that were as large as those from control GFP-expressing neurons. But blocking all activity in the network by rearing the animals in TTX restored normal arbor size to the Kir2.1-expressing cells, indicating a competition based on overall activity levels can also regulate arbor elaboration. Electroporation to express Kir2.1 in RGCs in mice, produces a similar result, with axon arbor elaboration greatly reduced compared to control cells (Benjumeda et al., 2013).

Interestingly, partial ablation of the SC at birth in the Syrian hamster results in an enhancement in the steepness of the ephrin-A gradient in the remaining part of the colliculus that is consistent with the accompanying compression of the retinotectal map (Tadesse et al., 2013). Implantation of a slow-release Elvax polymer to deliver the NMDAR blocker amino phosphonovaleric acid (APV) to the SC during development fails to prevent map compression in response to partial SC ablation, as measured electrophysiologically (Huang and Pallas, 2001). Thus the compression of the map appears to depend more upon molecular than on activity-dependent cues. However, receptive field sizes are enlarged in these APV-treated animals, consistent with neural activity playing an important role in map refinement. Thus, the experimental evidence indicates that the rough retinotopic mapping of axon arbors, as well as map compression and expansion, is largely the result of activity-independent mechanisms, including guidance molecule expression and competition for space in the overall target structure. Axon arbor size, important for the precision of connectivity, is regulated by activity-dependent competitive interactions.

## 3. Axonal and Dendritic Arbors Are Highly Dynamic, Even After Seemingly Mature Morphology Is Attained

The remarkable potential for structural plasticity observed in the developing and regenerating retinotectal projections reflects the cellular mechanisms by which retinal axons ramify within the optic tectum. Static images of labeled cells, reconstructed from fixed histological specimens, reveal convoluted trajectory changes and the frequent presence of interstitial branches throughout the axonal arbor which hint at the fact that axon growth and arbor development result from a highly exploratory process involving extensive axon remodeling over time (Sakaguchi and Murphey, 1985; Nakamura and O’Leary, 1989; Cline and Constantine-Paton, 1990; Dhande et al., 2011). However, live imaging of axonal and dendritic remodeling in intact, transparent zebrafish and *Xenopus* embryos has revealed a far more dynamic reality in which axons are perpetually extending and retracting extensive interstitial branch tips to probe the target area (O’Rourke and Fraser, 1990; Kaethner and Stuermer, 1992; Dingwell et al., 2000). In zebrafish, the process by which an axon arrives at and elaborates extensive branch tips within its final termination zone is not directed growth as one might find with chemoattraction, but rather what appears to be a process of random branch extension in which the overall progression of branch elongation and stabilization favors the future termination zone (Kita et al., 2015). The process is similar in *Xenopus* except that individual arbors occupy a relatively larger proportion of the total tectal neuropil from earlier stages, creating a situation in which the topographic map increases in precision with age, not only by restricting axonal branches to appropriate locations, but also by constant growth of the total retinorecipient field with age (Sakaguchi and Murphey, 1985). As the tectum expands by adding cells at its caudomedial pole, the RGC arbors adjust and improve their relative retinotopic order by gradually shifting their positions within the tectum. This creates a need for ongoing structural dynamism and plasticity at least until metamorphosis in order to optimize the map. The dendritic arbors of tectal neurons and even the filopodial processes extended by radial glial cells are similarly labile during this period, consistent with the notion that dynamic process remodeling can combinatorially increase the potential set of connections available for the network to sample and also reduce the steric interference that may result when multiple cells are actively rewiring within the same volume (Rajan et al., 1999; Chklovskii et al., 2004; Tremblay et al., 2009). Thus even in relatively mature tadpoles, in which most RGC axons have attained their mature size and complexity, time lapse imaging still reveals ongoing remodeling and exploratory probing at branch tips, albeit at considerably slower rates than are observed during the initial establishment of the retinotectal projection.

## 4. Patterned Neuronal Activity Provides Instructive Cues That Help Refine Inputs

The relative contributions of molecular signaling vs. neuronal activity in topographic map establishment and refinement have long been a subject of debate. The apparent lack of a requirement for action potential firing in the initial establishment of retinotopy has renewed questions about the overall importance of activity in map formation (Harris, 1984; Stuermer et al., 1990; Benjumeda et al., 2013). In this section, we have made an effort to describe the various experimental approaches, covering nearly a half-century, that have contributed to the conclusion that patterned neuronal activity is indeed instructive for precise retinotopic map refinement. We further discuss recent efforts from *in vivo* imaging to dissect how specific properties of patterned neuronal activity instruct different aspects of retinotectal refinement.

### Effects of Dark Rearing

Dark-rearing repeatedly has been shown to have no significant impact on topographic precision in the retinotectal projections in fish or amphibians, measured either electrophysiologically or morphologically. In the optic tectum of *Xenopus* frogs, dark-rearing produced no significant modifications in multiunit tectal receptive field sizes or in the laminar segregation of RGC inputs defined by specific stimulus selectivities (Keating et al., 1986). There are also no obvious alterations in the proper laminar targeting of RGC inputs within the superficial layers of the optic tectum in dark-reared compared to control zebrafish (Nevin et al., 2008). Furthermore, visual deprivation following optic nerve crush in adult goldfish does not impair the gradual sharpening of the initially diffuse termination field of regenerating retinal afferents into fine patches (Olson and Meyer, 1991). At first glance, these data appear consistent with the possibility that visual experience, and patterned neural activity, may not play a meaningful role in directing RGC axons to refine their projections to topographically appropriate tectal partners, and that the precise spatial organization of inputs in the tectum is exclusively determined by graded molecular guidance cues. However, it is critical to bear in mind that dark rearing does not necessarily deprive the visual system of all activity as spontaneous activity may be sufficient to provide the necessary activity-dependent cues needed for normal retinotopic map refinement. Work in the mammalian visual system certainly suggests that the maintenance of receptive field properties in the SC requires ongoing patterned visual input (Carrasco et al., 2005).

### Blockade of Action Potential Firing

In contrast to dark-rearing, experiments using chronic pharmacological blockade of voltage-gated sodium channels with TTX can directly test the requirement for action potential firing in development. Schmidt and Edwards (1983) reported that, unlike with dark-rearing, intraocular injection of TTX during optic nerve regeneration in adult goldfish prevented the refinement of multiunit receptive field sizes in the TTX-treated animals. It also resulted in the degradation of precision in the anatomical projection (Meyer, 1983). At the single cell level, TTX treatment resulted in significantly enlarged regenerated axonal arbors, but failed to induce any detectable alterations in an intact projection (Schmidt and Buzzard, 1990).

In *Xenopus laevis* tadpoles, retinal action potential blockade with TTX leads to a rapid increase in axonal branch dynamics measured as number of branches added and lost per 2 h (Cohen-Cory, 1999). The TTX-treated arbors also undergo greater net growth and branch addition over 24 h. In zebrafish larvae, however, the size and topographic location of individual RGC terminal arbors is not altered when action potential firing is blocked and in macho mutant fish with reduced sodium channel activity during development, perhaps reflecting the relatively faster pace of development in this species (Stuermer et al., 1990; Gnuegge et al., 2001). Interestingly, however, both TTX treatment and the macho mutation result in greater divergence of the retinotectal axons as they project into the tectum from the same quadrant of the eye, suggesting that while individual axon arbors are morphologically normal, they fail to converge precisely within their correct termination zone. In the mouse, silencing RGCs by *in utero* electroporation of Kir2.1 also does not prevent axonal pathfinding or targeting in the SC, but this manipulation does result in less elaborate, more diffusely arborizing axon terminals, indicative of a degraded retinocollicular map (Benjumeda et al., 2013).

### Blockade of NMDA Receptors

Locally-correlated, patterned firing in the retina, whether mediated by visual stimuli or spontaneous retinal waves, carries information about the relative locations of RGCs with respect to one another that the system can use to instruct map refinement. The notion of correlated firing between pre- and postsynaptic cells leading to strengthening of connection efficacy was originally articulated by Canadian psychologist Hebb (1949) in the context of learning and memory. The idea of a “Hebb synapse” capable of modifying its synaptic strength in response to co-activation of pre- and postsynaptic partners is in fact born out by the basal occlusion by Mg^2+^ of the ion channel of NMDA receptors, the principal glutamate receptor type found at newly formed synapses. Only when the dual requirements of glutamate binding and simultaneous postsynaptic depolarization to relieve the Mg^2+^ block of the pore are satisfied can the NMDAR flux current (Nowak et al., 1984). This property of the NMDAR means that it can function as a molecular detector of correlated activity. The fact that NMDAR activity appears to be required for many forms of neural plasticity, including long-term potentiation of excitatory synaptic transmission is consistent with this role.

One of the first demonstrations that correlation detection by NMDARs likely contributes to retinotectal map refinement involved the implantation of a slow-release polymer to continuously deliver the NMDAR antagonist APV over the optic tectum in Rana pipiens frogs and tadpoles. After several weeks of tectal NMDAR blockade a focal injection of a retrograde tracer was made into the optic tectum in order to reveal the convergence of inputs from the eye, a measure of map refinement. Compared with sham treated control animals, the animals that had undergone tectal NMDAR blockade exhibited a pronounced degradation of input convergence resulting in less precise retinotectal maps (Cline and Constantine-Paton, 1989). A similar experiment performed on early postnatal rat SC gave consistent results, leading to many mistargeted retinocollicular axon terminals and disrupting the normal refinement of termination zones of RGC axons (Simon et al., 1992). Electrophysiological analysis of SC receptive fields in the Syrian hamster provided functional evidence that NMDAR blockade during development also prevents the normal refinement of receptive field size (Huang and Pallas, 2001). Thus, Hebb’s postulate that “cells that fire together, wire together” indeed appears to be implemented by NMDARs, presumably acting as correlation detectors.

### Dually Innervated Tectum

The normal retinotectal projection in fish and frogs is almost exclusively contralaterally projecting and monocular. Surgical ablation of one of the tectal lobes or surgical deflection of the optic fibers from one tectal lobe to the other forces both eyes to map onto a single lobe, resulting in a dually innervated tectum (Sharma, 1973; Levine and Jacobson, 1975; Springer and Cohen, 1981). Alternatively implanting a supernumerary third eye during embryogenesis results in a projection that must share the optic tectum with the animal’s normal retinal inputs (Constantine-Paton and Law, 1978). The retinal afferents in dually innervated tectal lobes segregate into alternating ocular dominance bands, each dominated by inputs from one eye (Higenell et al., 2012). Because the tectum is normally monocular, it seems unlikely that any cryptic molecular patterning exists to program the segregation of retinal afferents into ocular dominance bands. Instead, it has been proposed that ocular dominance bands in the dually innervated tectum reflect a compromise between Sperry’s chemoaffinity cues and a Hebbian convergence of co-active inputs. Presumably each eye expresses the full complement of graded molecular guidance cues and therefore their axons seek to target topographically appropriate sites in the optic tectum. When two such axons from the same eye terminate in the same part of the tectum, Hebbian mechanisms should facilitate their convergence, but in the case where two axons from different eyes attempt to terminate in the same location they may be forced apart by competitive mechanisms as a consequence of their poorly correlated firing patterns. Evidence that neuronal activity indeed mediates the segregation into ocular dominance bands comes from experiments in which the firing of action potentials in retinal axons in 3-eyed frogs was chronically blocked by TTX, which resulted in the desegregation of inputs into a uniform, overlapping field in the tectum (Reh and Constantine-Paton, 1985). Moreover, the activation of tectal NMDARs specifically is also required, as chronic delivery of APV from a slow-release polymer placed over the tectum also desegregated the afferents from both eyes (Cline et al., 1987). Though it remains formally possible that neural activity and tectal NMDAR activation are merely permissive for segregation into ocular dominance bands, at least no band-like pattern of ephrin-A expression in the tectum, which would foreshadow the formation of eye-specific bands, has been observed in the dually innervated tectum (Higenell et al., 2012).

### Stroboscopic Rearing

Further evidence for activity patterns being instructive rather than merely permissive for activity-dependent retinotectal projection refinement comes from strobe-rearing experiments, in which an atypically high degree of correlation in the firing activity of RGCs is induced across the entire eye. The retinotectal projections in goldfish reared by stroboscopic illumination after hatching overlap substantially and fail to refine throughout development (Schmidt and Buzzard, 1993). Labeling of single RGCs in strobe-reared fish revealed axonal arbors that are long and diffusely branched without forming the characteristic dense clusters of branches at the termination zone multiunit receptive field maps in these animals also showed poor topographic refinement, with atypically large response fields. Regenerating projections exhibit a similar failure to refine under conditions of stroboscopic illumination (Schmidt and Eisele, 1985). Interestingly, the effects of strobe rearing on ocular dominance bands in the dually innervated fish or frog tectum has not to our knowledge been reported, though differences in axonal path length from the two eyes could produce sufficiently asynchronous synaptic activation in the tectum to reinforce segregation. On the other hand, a study in mice, which normally do have binocular innervation of the SC, found that in animals that had experienced optogenetic simultaneous co-activation of the two eyes during the period of retinotectal axon ingrowth prior to eye-opening, ipsilateral eye afferents were no longer restricted to deeper tectal layers but instead appeared able to stabilize inputs within the more superficial layers where contralateral inputs normally terminate exclusively (Zhang et al., 2011).

## Hebbian Mechanisms: Synchronous Firing Stabilizes Synapses and Prolongs Branch Lifetimes While Actively Suppressing Branch Dynamics Via Nmdar-Dependent Retrograde Signaling

The amenability of the intact *Xenopus* tadpole retinotectal system to live imaging and whole-cell electrophysiology makes it an ideal system in which to ask questions about the fundamental cellular events that underlie Hebbian structural plasticity. The retinotectal synapse was one of the first synapses shown to exhibit spike-timing-dependent long-term potentiation (tLTP) and depression (tLTD) *in vivo* (Zhang et al., 1998; Tsui et al., 2010). During the period of retinotectal map refinement, repeated visual stimulation can be used to induce conditions favorable for tLTP and this results in a shift in receptive field structure toward the potentiated subfield (Vislay-Meltzer et al., 2006). As in many other brain areas, tLTP in the retinotectal system is pathway-specific and NMDAR-dependent. One advantage of tLTP as a model for experience-dependent plasticity is the fact that it is far more physiological than protocols like tetanic stimulation and therefore more likely to resemble the actual mechanisms by which sensory input and patterned activity alter synaptic strengths in the developing visual system. As in the hippocampal Schaffer collateral-CA1 synapse, induction and expression of retinotectal LTP are both mediated by signaling events in the postsynaptic cell, which involves activation of NMDARs, Ca^2+^ influx, activation of Ca-calmodulin kinase type II (CaMKII), and trafficking of α-amino-3-hydroxy-5-methyl-4-isoxazolepropionic acid type glutamate receptors (AMPARs) to the synapse (Wu et al., 1996; Mu and Poo, 2006). In order for these phenomena to be relevant to map refinement, however, such postsynaptic signaling must be able to drive changes in the presynaptic axons through the production of one or more retrograde signals that can act back on the presynaptic terminal.

Normal visual experience during the period of developmental refinement can activate postsynaptic NMDARs. Indeed, blockade of NMDARs by bath application of APV results in a rapid upregulation of presynaptic RGC axon branch dynamics visualized *in vivo* by confocal microscopy, with a greater number of new branch tips added and retracted at the axon terminal over minutes to hours (Rajan et al., 1999). Application of the non-competitive NMDAR blocker MK-801 in zebrafish at 3 days post-fertilization when the retinotectal projection is first established is reported to result in an overall expansion of RGC axon arbor size (Schmidt et al., 2000). However because NMDARs are present not only in postsynaptic tectal neurons, but also in the retina and potentially at presynaptic terminals of RGCs in the tectum (Corlew et al., 2008; Banerjee et al., 2016) pharmacological blockade of NMDARs is not conclusive evidence for the existence of retrograde signaling.

More conclusive evidence for a retrograde signal originating in the postsynaptic cell that can modify the growth of presynaptic axons comes from an impressive series of *in vivo* time lapse imaging studies in *Xenopus* tadpoles by Zou and Cline (1996). Viral overexpression in tectal neurons of a constitutively active truncated form of CaMKII (tCaMKII), which lacks the autoinhibitory regulatory domain, mimics the activation of CaMKII that takes place in LTP induction. Animals in which the postsynaptic tectal neurons, but not the presynaptic RGCs, were virally infected with tCaMKII showed the expected enhancement in synaptic AMPAR currents as NMDAR-only “silent synapses” matured *en masse* to become AMPAR-containing functional synapses (Wu et al., 1996). Interestingly, the RGC axon arbors were visualized in these animals and found to grow far less and exhibit much lower branch tip density than control cells, indicating the existence of a retrograde signal downstream of CaMKII activation that stabilizes existing branches and suppresses branch elaboration as it drives synaptic maturation.

The process of ocular dominance band formation in dually innervated fish and frog tectum (described above) almost certainly requires retrograde signaling, as the correlation detection is most likely performed by NMDAR activation in postsynaptic neurons. At the level of single axon branch dynamics, time-lapse imaging of *Xenopus* RGC axon arbors in dually innervated tectum reveals a preferential stabilization of branches that extend into same eye territory, compared to territory dominated by the other eye (Ruthazer et al., 2003). This preference is eliminated when NMDARs are pharmacologically blocked, a result that conforms with the idea that NMDARs mediate axon branch stabilization via retrograde signaling.

## Stentian Mechanisms: Asynchronous Activity Weakens Synapses (LTD) and Actively Promotes Axonal Branch Dynamics, Including Branch Addition, Elongation, and Elimination

To date, the most direct elucidation of how correlated firing among retinal afferents can instruct the refinement of the retinotectal map at the level of individual RGC axon branch dynamics has come from a study that took advantage of the fact that although the retinotectal projection in *Xenopus* tadpoles is almost purely contralateral, in the occasional animal one or two individual RGC axons can be found to project by accident to the ipsilateral optic tectum (Munz et al., 2014). These misguided axons arborize and form synaptic contacts within the ipsilateral tectum, presumably responding to the same molecular cues that guide the contralateral RGC axons to form a crude map. This creates a unique experimental system in which, by visually stimulating the two eyes independently and systematically varying the degree to which stimulation is correlated between the two eyes, it becomes possible to directly test the Hebbian “fire together, wire together” hypothesis. Because the contralateral eye drives most of the inputs, a flash of light presented to that eye will cooperatively recruit activation of postsynaptic tectal neurons. In contrast, for the lone ipsilateral RGC to participate in firing the tectal neurons, it must fire at the same time as the contralateral inputs.

In his 1973 treatise on Hebbian plasticity, Gunter Stent argued that there must exist a complementary rule to Hebb’s postulate to explain the case where a presynaptic axon repeatedly fails to excite a postsynaptic partner that is actively firing under the influence of another input (Stent, 1973). Stent proposed that this condition should be punitive, resulting in the weakening of that non-contributing input. This is sometimes referred to as the “Stentian extension” of Hebb’s postulate. The lone ipsilaterally projecting RGC axon allows for both Hebbian and Stentian forms of correlation-based plasticity to be examined by applying synchronous or asynchronous stimulation to the two eyes.

Electrophysiological recordings from tectal neurons that receive synaptic input from both the ipsilateral and contralateral eyes revealed that the ipsilateral input maintains or even slightly increases its synaptic strength relative to the contralateral inputs when both eyes are stimulated together. However, when the two eyes were stimulated 1 s apart, the ipsilateral eye input, which by itself is usually not strong enough to drive the postsynaptic neurons to fire action potentials, very rapidly declines in synaptic strength and in many cases entirely loses its ability to evoke an AMPAR-mediated postsynaptic current, suggesting that a phenomenon like tLTD can be induced by asynchronous visual stimulation of the two eyes in this case.

*In vivo* multiphoton time-lapse imaging of the misdirected ipsilateral axon was also performed while concurrently presenting these same synchronous or asynchronous visual stimuli to the two eyes. Remarkably, asynchronous stimulation resulted in a rapid (within 30 min) and dramatic upregulation of new branch additions and a significant increase in branch tip elongation compared with axon dynamics during a preceding period of darkness. Elimination of branch tips was also enhanced, indicating that rather than producing a larger arbor, asynchronous stimulation makes the axon more dynamic and exploratory. Thus, asynchronous visual stimulation produced an enhancement in growth and dynamics akin to the effects of NMDAR blockade. This makes sense as it is unlikely that the lone ipsilateral axon would by itself be able to drive sufficient depolarization of the postsynaptic tectal cell to permit Ca^2+^ flux through NMDARs. Consistent with this notion, addition of MK801 to block NMDARs did not prevent the increased rate of branch additions in response to asynchronous stimulation. It is therefore possible that the source of the branch promoting signal may not be postsynaptic in origin, but could, for example be released by surrounding glial cells or come directly from nearby axon terminals.

In contrast, synchronous stimulation of the two eyes resulted in a rapid decrease in the rate of branch additions to levels seen in darkness. This decrease in branch dynamic behavior was completely prevented in the presence of MK801, or if tetanus toxin was expressed in the ipsilateral axon to render it incapable of releasing neurotransmitter, indicating that the activation of postsynaptic NMDARs likely leads to the release of a retrograde branch suppressing factor. In addition, branches that did form during synchronous stimulation had longer lifetimes on average that those that emerged during periods of asynchronous stimulation, indicating that they were more stable overall.

**Figure 1:**
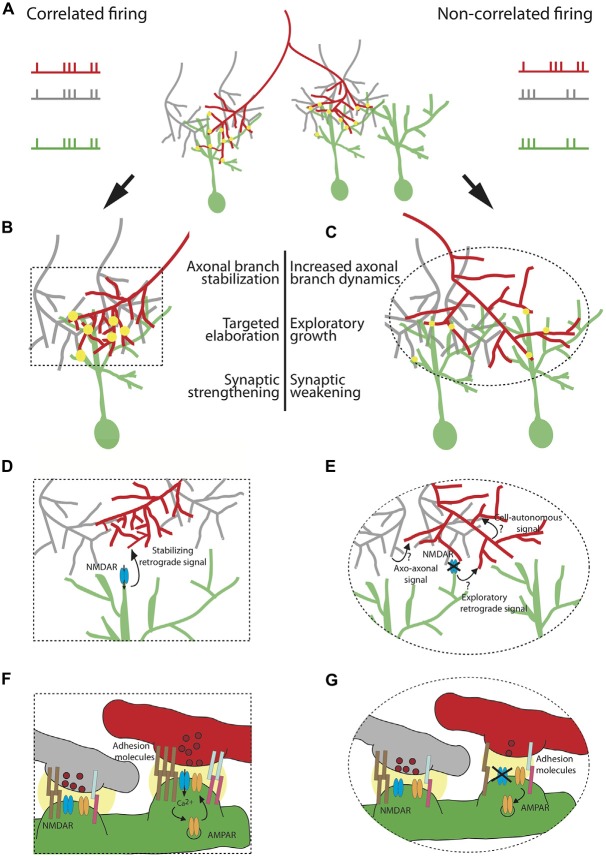
**Cellular and molecular mechanisms underlying the instructive role of patterned neuronal activity in retinotectal map refinement.** A retinal ganglion cell (RGC) axon of interest (red) synapses onto tectal neuron dendrites (green) in **(A)** The red axon (left branch) is co-active with its neighboring RGCs (gray) and its firing pattern is therefore correlated with the firing pattern of the tectal neuron on which it synapses. The right branch of the red axon is not co-active with its neighbors and its firing pattern is not correlated with the firing pattern of its synaptic partner. Synapses are depicted as yellow circles. Synaptic strength is represented by the size of the yellow circle. **(B)** Correlated firing patterns of an RGC with its partnering tectal neuron instruct an increase in synaptic strength and stabilization of the axonal arbor allowing for local targeted arbor elaboration as new branches tend to form at existing synapses. **(C)** Non-correlated firing of an RGC with its neighboring axons and its postsynaptic partner instructs synaptic weakening and an increase in axonal branch dynamics, accompanied by exploratory growth in search of a better partner. The effects of patterned neuronal activity on structural remodeling and synaptic efficacy are schematized in **(D–G)**, respectively. **(D)** Shows a zoom-in of the area of the box in **(B)** depicting a “stabilizing retrograde signal” downstream of activation of N-methyl D-aspartate receptor (NMDAR) which encodes axonal branch stabilization and targeted elaboration. **(E)** Zoom-in of the ellipse in **(C)** Plausible mechanisms instructing axonal exploratory growth and branch destabilization due to the lack of correlated firing with the neighboring RGC inputs and the postsynaptic partner include: 1. Axo-axonal signal, released by the firing neighboring inputs (gray); 2.“Exploratory retrograde signal”, unmasked by the inactivation of NMDAR; 3. Cell-autonomous activity-dependent signal, released by the red neuron or acting intracellularly. **(F)** Zoom-in depicting molecular mechanisms underlying synaptic strengthening and stabilization of the red axon from **(B)**. Correlated firing of the RGC axon (red) with its neighboring axons (gray) and the tectal neuron (green) results in both release of glutamate containing vesicles (dark red) and postsynaptic depolarization. This satisfies the conditions required for release of the Mg^2+^ block from the channel pore of the NMDAR (blue), allowing for cation influx. Ca^2+^ triggers a molecular cascade resulting in insertion of more AMPAR (orange) in the membrane. A retrograde signal downstream of NMDAR encoding higher probability of vesicle fusion in the red axon (depicted as higher number of vesicles in dark red). Increase in synaptic efficacy is accompanied by strong homophilic (brown) and heterophilic interactions of adhesion molecules (light blue and pink). The strength of the interaction is represented by the number of pairs of adhesion molecules. **(G)** Zoom-in showing the change is synaptic efficacy in **(C)**. Non-correlated firing of the red axon with its neighbors and its partnering tectal neuron prevents opening of the channel pore of NMDAR, leading to AMPAR endocytosis, lower probability of glutamate release and weaker interaction of adhesive molecules.

This experimental protocol tests the full range of growth responses that patterned activity might be able to induce, as it creates a set of extreme differences in firing correlation with synchronous stimulation resembling the conditions that might be produced by strobe rearing where all inputs are artificially correlated, and asynchronous stimulation creating a set of correlations that might only be found if the RGC were to ramify in an entirely inappropriate part of the tectum or what occurs in the dually innervated tectum. In the normal process of activity-dependent developmental refinement a typical axon might be expected to experience a more modest range of local correlation and asynchrony that would lead to a slight upregulation of exploratory branching and synapse disassembly on those branches that extend away from the proper termination zone (promoting them to keep growing until they land in more welcoming territory), and a stabilization and synaptic strengthening on those branches that extend into the appropriate part of the map where inputs with similar activity patterns converge (promoting consolidation and further synaptogenesis at this site). Figure 1 portrays several plausible models for how these mechanisms could promote projection refinement.

## 5. In the Absence of Sensory Input, Correlated Spontaneous Firing Provides Surrogate Patterned Activity

The pattern of action potential firing in the developing visual system contains information about the relative positions of the RGC somata in the retina and thus can instruct the precise mapping of the axons onto their postsynaptic partners in the optic tectum. Anamniotes, which include fish and amphibians, develop exclusively externally which allows for neuronal activity driven by the natural visual scenery (see review in this special topic issue by Pratt et al., 2016). Unlike fish and frogs, amniotes are hidden behind thick shells or develop *in utero*, which leads to a general deprivation of visual experience during the time when visual circuit refinement takes place. It is reasonable to speculate that spontaneous activity in the retina may have arisen as an evolutionary adaptation to provide replacement patterns in amniotes of neuronal activity that lower vertebrates are able to experience by visually interacting with their surrounding environment after hatching. The first evidence for locally correlated spontaneous activity in the fetal retinal came from extracellular retinal recordings made in rat pups while still attached to the uterus via the umbilical cord (Maffei and Galli-Resta, 1990). They have subsequently been confirmed and meticulously characterized using *in vitro* multielectrode array recordings and calcium imaging of retinal explants (Meister et al., 1991; Wong et al., 1993; Feller et al., 1996). These spontaneous activity patterns exhibits a high degree of local correlation in firing and consequently have been dubbed “retinal waves” (Meister et al., 1991). RGCs located in close proximity overlap their bursting activity in time, whereas RGCs that reside further away from each other are less likely to be co-active. This spatiotemporal pattern of RGC activity results from a local initiation of depolarization, which propagates to adjacent neurons spreading over long distances across the retina.

The definitive demonstration of this phenomenon was recently implemented in the intact mouse by loading RGCs out to their axon terminals with a calcium indicator to permit retinal waves in the eye to be detected by imaging their calcium transients in the SC (Ackman et al., 2012). This study revealed that the initiation point of the waves is biased to the ventrotemporal region of the retina, an observation that is particularly interesting in light of the fact that a locomoting fish or tadpole in the wild would typically experience natural visual stimuli as optic flow similarly sweeping from temporal to nasal retina, further arguing that retinal waves may have evolved as a replacement for natural vision prior to eye-opening. Retinal waves in zebrafish also stereotypically originate from the temporal retina (Zhang et al., 2016). In tadpoles, it has been shown directly that visual stimulation which includes optic flow in this more natural direction is far more effective at refining the retinotopic projection of RGCs than an identical amount of stimulation oriented in the opposite direction (Hiramoto and Cline, 2014). The mechanisms generating waves in the mammalian retina differ over development: embryonic type I waves depend on gap junctions; type II waves are initiated by starburst amacrine cells and spread through activation of nicotinic acetylcholine receptors; and type III waves utilize glutamate (Feller et al., 1996; Bansal et al., 2000; Torborg et al., 2005; see reviews in this special topic issue by Arroyo and Feller (2016) and Kerschensteiner (2016). In zebrafish, only one stage type of retinal waves has been described. They originate at the axonal terminals of bipolar cells and depend mostly on ionotropic glutamatergic receptors and gap junctions, with acetylcholine receptors likely having a modulatory role (Zhang et al., 2016).

While there is abundant evidence that patterned activity has an instructive role in topographic map refinement, an important remaining problem is to dissect the specific aspects of spontaneous activity that instruct retinocollicular refinement. Genetic deletion of the β2 nicotinic acetylcholine receptor subunit (β2-nAChR) in mice results in abnormal retinal waves with impaired spatiotemporal properties (Rossi et al., 2001; McLaughlin et al., 2003b; Chandrasekaran et al., 2005; Mrsic-Flogel et al., 2005). These perturbations result in severe defects in both the topographic refinement and eye-specific segregation in the SC and LGN. Unexpectedly, it was also reported that between-eye correlations in wave activity are enhanced in these β2-nAChR knock-out mice, which may partially account for the failure of eye-specific segregation (Burbridge et al., 2014).

Xu et al. (2011) examined a transgenic mouse line (β2(TG)nAChR) in which expression of the β2-nAChR was restored specifically in the RGCs in β2-nAChR knock-out animals. Although retinal waves in these mice occur with the same frequency and overall level of activity as in wild-type animals, their propagation is truncated and thus the correlation between the firing patterns of neighboring cells decreases steeply with distance. The very local correlations in RGC spiking are still intact, but the large-area within-eye correlations are lacking. These “smaller” retinal waves are sufficient to permit the refinement of retinotopy in the monocular zones of SC. Interestingly, however, RGC projections to the binocular zone fail to refine normally. This is apparently a result of an interaction between inputs from the two eyes, as monocular enucleation permits the full refinement of the remaining eye’s afferents. Eye-specific segregation is also strongly disrupted in these mice.

They also tested animals (Rxβ2-cKO) in which β2-nAChR expression was conditionally deleted specifically in the retina (Xu et al., 2015). Much like the β2(TG)nAChR mice, Rxβ2-cKO mice exhibit only small residual retinal waves that have high local correlation but much lower long-distance correlations in firing. Retinotopy in Rxβ2-cKO mice was normal in the monocular SC, but eye-specific segregation was disrupted, phenocopying the β2(TG)nAChR animals. The rescued retinotopy and impaired eye-specific segregation in the SC compared to full β2-nAChR knock-outs suggest that topographic precision of the visual map depends primarily on local correlation in spiking patterns, whereas eye-specific segregation requires global within-eye correlations that differentiate the two eyes. It remains to be tested whether the abnormal between-eye correlations seen in full β2nAChR knock-out mice might also occur in β2(TG)nAChR or Rxβ2-cKO lines. This binocular correlation could perhaps help explain the failure of RGC inputs to refine topographically in the binocular zone of the SC, as inappropriately correlated inputs would be converging on postsynaptic cells in the SC.

Spontaneous retinal waves occurring before the onset of vision have been observed across numerous amniote species: turtle, chick, rat, mouse, ferret, cat, and monkey (Ackman and Crair, 2014). A form of retinal waves have also been described in zebrafish (Zhang et al., 2016). However they do not appear to be present in amphibians, which are able to rely on photoreceptor-driven vision from the onset of development of the retinotectal projection.

Zhang et al. (2016) have shown that retinal waves in zebrafish are restricted to a very short developmental window 2.5–3.5 days post-fertilization (dpf). During this period RGCs begin to form synapses with postsynaptic tectal neurons (2 dpf) and visual behaviors such as prey capture and predator avoidance emerge shortly afterwards (4–6 dpf; Stuermer, 1988; Borla et al., 2002). Thus, these animals have a very short time to form a precise representation of the visual world necessary for survival. We can speculate that the available visually-driven and spontaneous activity might work in concert to provide adequate information for topographic map refinement (Zhang et al., 2010). Interestingly, Demas et al. (2012) used multielectrode arrays to record retinal activity across the eye, and discovered that rearing *Xenopus* tadpoles in complete darkness induces an increase in the amount of correlation in the spontaneous activity between neighboring RGCs. This observation supports the notion that the retinal circuitry may exhibit a tendency to favor retinal waves as a natural means of compensating for an absence of early visual stimulation. These critical discoveries finally helped make sense of several decades of earlier dark-rearing experiments in fish and frogs that had concluded that visual experience was dispensable for normal refinement of the developing retinotectal projection.

## 6. New Axonal Branch Tips Emerge Near Existing Synapses

At the same time as the retinotectal axons are dynamically remodeling by constant exploratory branch addition and withdrawal, fish larvae and tadpoles are already using their visual system to interact with the environment. Zebrafish are avid predators that can track and capture tasty paramecia and other organisms for food, a behavior that requires precise tectal function (Gahtan et al., 2005). This raises the paradox of how a functional circuit can at once be wired to reliably perform essential behavioral tasks while actively adding and eliminating synaptic contacts to refine connectivity. Insights into this process have come from time-lapse imaging of RGCs axons and tectal neurons expressing fluorescently labeled synaptic marker proteins to reveal synapse locations. This powerful approach was pioneered in the retinotectal system by Alsina et al. (2001) who expressed GFP-VAMP2 in *Xenopus* RGCs to reveal that many putative synaptic sites along the developing axonal arbor are added and eliminated rapidly over time, the rate of which can be regulated by Brain-Derived Neurotrophic Factor (BDNF) signaling. These authors made the important observation that new axonal branch tips almost always emerged from GFP-VAMP2 positive puncta, a finding that was later confirmed using a better targeted synaptic marker, synaptophysin-GFP (Meyer and Smith, 2006; Ruthazer et al., 2006). This result has profound implications as a mechanism for map refinement because it means that wherever a synapse strengthens (or weakens) through activity-dependent plasticity, it will be available (or not) to nucleate new branches from which new synapses can form. This constitutes a positive feedback loop that will lead to the targeted elaboration of axonal arbor at sites where that axon has formed effective, strong synaptic contacts and the scaling back of branch initiation at inappropriate sites where synapses may form transiently but are subsequently eliminated.

## 7. Stronger Synapses Help Stabilize the Axons and Dendrites on Which They Reside (Synaptotropism)

Postsynaptic dendritic growth and remodeling was studied in zebrafish tectal neurons co-expressing PSD-95-GFP to mark postsynaptic sites (Niell et al., 2004). These investigators made the critical observation that synaptic sites were fairly labile. They observed that the dendritic tree elaborated through a process of dynamic filopodial extensions followed rapidly by synapse formation. As synapses formed, those synapse-bearing branches became consolidated. Further branch extension then proceeded by building upon these more stable sites. Thus the presence of a synapse appears to confer stability onto the branch on which it forms, a phenomenon referred to as “synaptotropism” (Vaughn et al., 1988; Cline and Haas, 2008). Further support for the synaptotropic model of dendritic growth was solidified by experiments where synaptogenesis or synapse maturation respectively were prevented by blocking neurexin/neuroligin signaling or AMPAR trafficking in *Xenopus* tectal neurons, resulting in a failure to elaborate normal complex dendritic arbors (Haas et al., 2006; Chen et al., 2010).

Time lapse imaging of dsRed/synaptophysin-GFP-expressing RGC axons in zebrafish and *Xenopus* tadpoles showed that synaptotropism is equally applicable at the presynaptic side (Meyer and Smith, 2006; Ruthazer et al., 2006). Within minutes of axonal branch extension, synaptophysin-GFP puncta could be observed accumulating in the wake of the advancing growth cone. Some synaptic puncta were later lost while others became more mature over time, indicated by the bright accumulation of synaptophysin-GFP positive vesicles. When these branches later attempted to retract, the presence of a mature synaptic site conferred structural stability, preventing the branch from withdrawing beyond that site.

## 8. Homeostatic Mechanisms Help Maintain the Overall Level of Functional Synaptic Input to the Target

Both the Hebbian and Stentian mechanisms in the context of changes in synaptic efficacy are inherently unstable. Correlated firing between a presynaptic neuron and its postsynaptic partner would induce synaptic strengthening as described above. An increase in synaptic efficacy would result in an even higher probability of correlation between the firing patterns of the pre- and postsynaptic neuron. Thus, applying only the Hebbian plasticity rules, the positive feedback loop would become unsustainable. Applying the same logic to Stent’s extension to the Hebbian postulate, we will find ourselves in a similar situation where each time the synapse weakens, it will be less likely that the pre- and the postsynaptic firing patterns will be correlated. The brain overcomes this inherent instability by applying additional rules which ensure a healthy dynamic range of synaptic transmission within which bidirectional changes in synaptic efficacy can occur. These rules are referred to as homeostatic plasticity and have been extensively studied in the neocortex and the hippocampus (Bienenstock et al., 1982; Turrigiano and Nelson, 2004; Kaneko et al., 2008). An example of homeostatic regulation in the developing *Xenopus* retinotectal projection can be found in experiments where the intrinsic excitability of tectal neurons was manipulated either by overexpression of leak K^+^ channel or by modifying synaptic efficacy by application of a peptide that hinders AMPAR trafficking. Both manipulations lead to upregulation of voltage-gated Na^+^ currents (Pratt and Aizenman, 2007), suggesting a homeostatic mechanism regulating intrinsic excitability that counteracts Hebbian/Stentian plasticity rules.

Further evidence for homeostatic regulation of the retinotectal circuit during development was obtained using zebrafish *blumenkohl* mutants (*blu*). The *blu* mutation disrupts vglut2a, encoding a vesicular glutamate transporter homologous to the mammalian VGLUT2 (Smear et al., 2007). *Blu* mutants exhibit a decrease in TTX miniature EPSC (mEPSC) amplitudes in tectal neurons, suggesting a reduction in glutamate concentration per vesicle. Interestingly, mEPSC frequency in mutant animals is increased, consistent with a higher probability of glutamate vesicle release or with upregulation in the number of release sites. These observations allude to a compensatory mechanism that helps normalize glutamatergic transmission in these animals. In accordance with this homeostatic regulation, the RGC arbors in the *blu* mutant zebrafish are larger, spanning a greater area of the optic tectum and tectal neurons exhibit larger receptive fields. In tectal *blu* animals neurotrophin-3 (NT-3) protein levels are upregulated in the optic tectum, suggesting that it could act as a homeostatic retrograde signal through TrkC receptor promoting axonal branch elaboration (Auer et al., 2015).

## Summary

**Figure 2:**
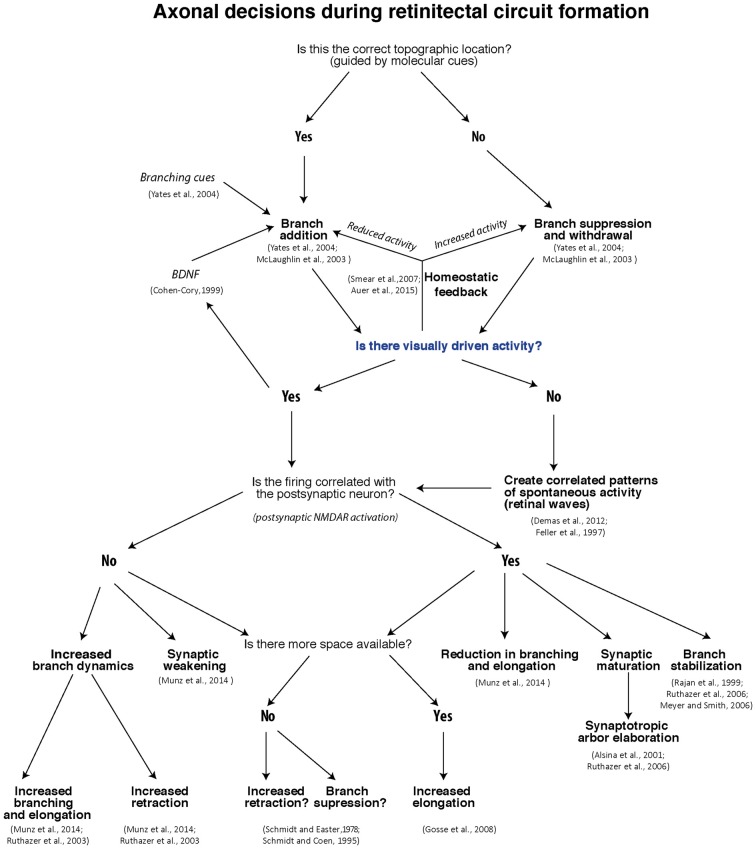
**Flow-chart of decisions RGCs face during retinotectal map formation and refinement**.

Thanks to many decades of experimentation on retinotectal development in models ranging from fish to mammals, in conjunction with modern technology permitting live imaging of developing axons in the intact animal, we now have a much clearer understanding of the mechanisms that regulate the developmental fine-tuning of the retinotectal map. The decisions faced by a growing retinotectal axon are summarized in the form of a flow chart in Figure 2. While it is likely that other brain regions will apply slightly different strategies for activity-dependent refinement, the rules we have outlined here should prove a useful template for further investigation.

## Author Contributions

This article was written jointly by EK and ESR in consultation with MM. Figures were made by EK and MM.

## Funding

EK holds a Jeanne-Timmins Costello Fellowship from the Montreal Neurological Institute. MM holds a Human Frontiers Fellowship. ESR holds a Fonds de Recherche du Québec - Santé Research Chair.

## Conflict of Interest Statement

The authors declare that the research was conducted in the absence of any commercial or financial relationships that could be construed as a potential conflict of interest.
